# PESCADOR, a web-based tool to assist text-mining of biointeractions extracted from PubMed queries

**DOI:** 10.1186/1471-2105-12-435

**Published:** 2011-11-09

**Authors:** Adriano Barbosa-Silva, Jean-Fred Fontaine, Elisa R Donnard, Fernanda Stussi, J Miguel Ortega, Miguel A Andrade-Navarro

**Affiliations:** 1 Computational Biology and Data Mining Group, Max Delbrück Center for Molecular Medicine. Robert-Rössle-Strasse. 10, D-13125, Berlin, Germany; 2 Laboratório de Biodados, Dpto. de Bioquímica e Imunologia, ICB - UFMG. 31270-901, Belo Horizonte - MG, Brazil; 3 São Paulo Branch, Ludwig Institute for Cancer Research, 01323-903, São Paulo - SP, Brazil

## Abstract

**Background:**

Biological function is greatly dependent on the interactions of proteins with other proteins and genes. Abstracts from the biomedical literature stored in the NCBI's PubMed database can be used for the derivation of interactions between genes and proteins by identifying the co-occurrences of their terms. Often, the amount of interactions obtained through such an approach is large and may mix processes occurring in different contexts. Current tools do not allow studying these data with a focus on concepts of relevance to a user, for example, interactions related to a disease or to a biological mechanism such as protein aggregation.

**Results:**

To help the concept-oriented exploration of such data we developed PESCADOR, a web tool that extracts a network of interactions from a set of PubMed abstracts given by a user, and allows filtering the interaction network according to user-defined concepts. We illustrate its use in exploring protein aggregation in neurodegenerative disease and in the expansion of pathways associated to colon cancer.

**Conclusions:**

PESCADOR is a platform independent web resource available at: http://cbdm.mdc-berlin.de/tools/pescador/

## 1. Background

The repository of biomedical literature available from the NCBI's PubMed database [1] is used by researchers to find references related to particular topics or authors. This resource contains a wealth of biological data but it is vast (currently containing more than 20 million records) and therefore multiple tools have been generated to search it (recently reviewed in [2]). On the one hand, thematic analysis within biomedical text has been used to arrange bibliography according to topics in clusters [3,4] or categories [5], or to find literature relevant to genes [6,7]. On the other hand, PubMed is a formidable resource for information extraction tools, for example to obtain references to genes [8], relations between genes [9], functional gene annotations [10] or gene associated bibliographic profiles [11].

A particularly valuable task in information extraction is the identification of biomolecular interactions from biomedical text data where the interactors and the type of interaction are identified [12,13], for example, a protein-protein interaction (PPI) between Neuroserpin and Abeta. Some web tools such as iHOP [14], STRING [15] or AliBaba [16], can generate networks that include biomolecular interactions extracted from the literature.

However, current text mining tools for biomolecular interactions are not flexible enough to filter interactions extracted from a thematic PubMed query (centered on a novel research theme of interest) according to concepts considered significant for the query. For example, finding PPIs related to protein aggregation, as in "BRI2 inhibits Abeta aggregation", or relevant to disease as in "Neuroserpin binds Abeta and is a neuroprotective component of amyloid plaques in Alzheimer disease."

The LAITOR tool [17] was developed to fill this gap as an original text-mining strategy that allows user-defined biological concepts to be searched along the co-occurring bioentities. However, the method was implemented as a MySQL-dependent PHP command-line script, which makes it difficult to biologists lacking computer skills to use the system as it requires the installation of software and specialized term dictionaries, lacks tools to explore and display the evidence behind the extracted information, and does not include mechanisms to share and annotate data relevant for a given user.

To expand the applicability and functionality of the text-mining method implemented in the LAITOR tool, we have developed PESCADOR (**P**latform for **E**xploration of **S**ignificant **C**oncepts **A**ssociate**D **to co-**O**ccurrence **R**elationships), an online tool that allows users to input their own selections of abstracts and protein interaction related concepts, to extract interactions between pairs of biomolecules, select them by type or interacting partners, and visualize them graphically as a network. PESCADOR uses pre-compiled dictionaries of terms (from Entrez Gene [18] and UniProt [19]) for every organism with deposited genes (NCBI Taxonomy Database [1,20]) and dictionaries of biological concepts (Medical Subject Headings, MeSH). Therefore, biologists need to simply load (copy/paste) their literature of interest (a list of PubMed identifiers, PMIDs) to launch the text-mining analysis.

Contrary to other web tools mentioned above that represent literature-derived biomolecular interactions, PESCADOR focuses on flexible inputs and outputs and in the representation of the network of interactions and related concepts. Such a resource is different from iHOP, which does not generate a network view, from STRING, which does not use PubMed abstracts as input, and from AliBaba, which does not allow the user to input selections of PubMed abstracts or concepts. In this respect, PESCADOR constitutes a resource that is complementary to these other tools.

## 2. Implementation

### 2.1. System architecture

PESCADOR is an online resource developed using the PHP programming language (version 5.3.2). A user query is uploaded by an HTML form. This query is composed of a list of PMIDs to be scanned for gene/protein co-occurrences and, optionally, of a list of words (ideally, biological concepts related to protein interactions, such as "aggregation" or "phosphorylation") to be found in the co-occurrence analysis. The list of PMIDs can be either typed, provided in a file, or obtained by a query to NCBI's PubMed [1], MedlineRanker [21] (see button "Send All" in the "Send results to Pescador co-occurrence analysis tool" section in MedlineRanker's output page) or XplorMed [4] (see button "Send to PESCADOR" in XplorMed's output page). Next, the query is assigned a process ID and loaded on a job list. A launch agent reads the job list every two seconds and selects queuing processes to be executed. Finally, the selected process is subject to text mining analysis (see next sections), which includes tagging the requested PubMed abstracts. Tagged abstracts are stored in a local database to save time for future searches. When finished, a script adds the ID of the finished process on a list of completed jobs, whose results can be browsed or downloaded by users within 30 days from the run.

### 2.2. Text mining using LAITOR

PESCADOR uses LAITOR [17] as text-mining engine to extract sentences with co-occurring bioentities (genes and proteins) from the text of the PubMed abstracts requested. First, LAITOR uses the NLProt program as information extraction tool [22] to tag the abstracts for bioentities using a species-specific dictionary composed of symbols and synonyms for the genes/proteins of the organism selected by the user, which includes non-redundant names and alternative names from the corresponding UniProtKB records [19]. Next, LAITOR identifies biointeraction terms in the text of the abstracts according to a dictionary of biointeraction terms. Finally, co-occurrences between the previously identified bioentities are classified in four types (from less to more significant [17]): two bioentities co-occur in abstract (type 4), they co-occur in sentence (type 3), they co-occur in a sentence with a biointeraction term (e.g. activates, induces, inhibits) anywhere in the sentence (type 2) or co-occur in a sentence with a biointeraction term in between the bioentity names (type 1). In the current implementation of PESCADOR, sentences containing four or more bioentities are excluded from the analysis since they tend to be too complex to automatically extract interactions.

LAITOR does not handle negations, e.g. a sentence such as "protein A does not bind B". One possibility is to try to recognize these sentences in order to just avoid them as it is difficult to identify if the negation refers to the information being extracted (see for example [23]). However, the number of sentences negating a biological fact found in abstracts is small and a pragmatic approach is to deal with them as with any other sentence under the assumption that this produces a small number of false positives (see for example [24]).

A detailed description of LAITOR, including a standard benchmarking against the BioCreative II IAS dataset, can be found in the original publication [17].

### 2.3. Definition of concept dependencies

If the user provides a list of concepts (phrases composed of one or more words, which are meaningful for the user), those will be used to evaluate co-occurrence between those concepts and the previously extracted bioentities. First, the text of the abstract is scanned for occurrences of the phrases present in the list of concepts. Then, bioentity co-occurrences within a sentence (types 1-3) are associated to concepts found in the sentence, and anywhere-in-the-abstract bioentity co-occurrences (type 4) are associated to all concepts found in the abstract.

### 2.4. Website structure

The PESCADOR website is organized as an input HTML form and subsequent pages that permit users to navigate on completed analyses. These pages are described below.

Home page: in this page users can load a PMID list from a file, type it manually, or obtain it from a query to PubMed, MedlineRanker or XplorMed as explained above. In addition, a list of concepts of interest (e.g. "aggregation", "brain") can be optionally loaded. Alternatively, previously analyzed projects can be retrieved from our system by their process ID.

Status page: in this page, the status of the abstracts' retrieval, tagging and co-occurrence analysis are shown. Once all processes are finished, a link to the summary page is exhibited. Otherwise, a progress message is displayed.

Summary page: this session displays the results available and is composed of two sub-sessions: browse and download results.

Terms page: shows a list with all terms identified in the co-occurrence analysis represented with a variable font size that increases with the number of abstracts where the term was found. Once a term is selected, it is displayed in a table with the Gene ID mapped to that term, and the UniProtKB terms mapped to this gene. Duplicated tables are shown for ambiguous terms. Furthermore, a table with the co-occurrences for the selected term is also displayed, where it is possible to verify the pair, biointeractions list, types of co-occurrences (from type 1 to 4, as defined by LAITOR, see Methods) and abstract's sentences and PMIDs from which the pair has been extracted.

**Figure 1:**
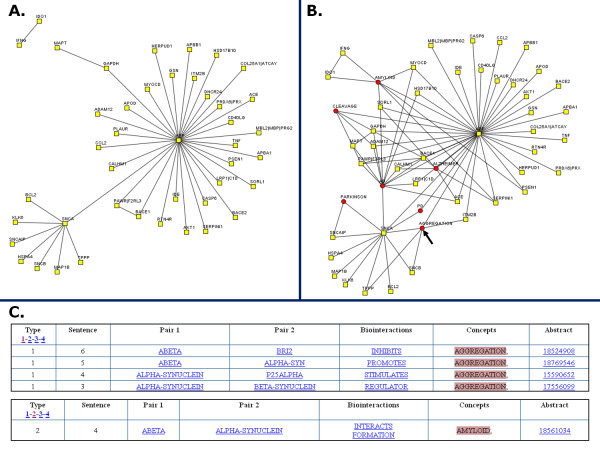
**Example of sub-network merging by PESCADOR using biological concepts**. (A) Co-occurrence network created using 49 abstracts related to Alzheimer and Parkinson diseases (available at PESCADOR website). The network display has been filtered to show only co-occurrences of type 1 (highly confident). (B) Pushing the 'Show concepts' button adds concepts to the interaction network, which allows relating parts of the network to properties such as "AGGREGATION" (black arrow). (C) Filtered co-occurrences where the concept "AGGREGATION" has been found to connect terms related to Parkinson Disease (P25ALPHA, ALPHA-SYNUCLEIN, BETA-SYNUCLEIN) to those of Alzheimer Disease (BRI2 and ABETA).

Concepts page: shows the list of concepts found from the list that was loaded, with their respective co-occurring pairs, pointers to the source abstract (Figure 1C), and a network of biointeractions from co-occurrences of genes with the selected concept (Figure 1B).

Abstracts page: shows the list with the loaded PMIDs. Once selected, an abstract text is displayed with the target sentence highlighted in green with violet for co-occurring bioentities, orange for biointeraction terms and blue for concepts. A table displays the entire set of co-occurring pairs extracted from the abstract. These pairs can be validated by the user by clicking in the button in the column "Validate pairs". This action will validate all instances of the pair associated to the given abstract and can be reverted.

Network page: shows a network generated by MEDUSA [25] inferred from the LAITOR co-occurrence analysis (Figure 1A). Terms are mapped by default to official gene symbols but the user can switch the display to raw terms. This page also shows a list with the terms and concepts present in the network, which can be linked to their respective report webpage. There is a control at the top, where users can select different parameters to be used to build the graph. We note that the applet displaying the network might be slow if many elements have to be displayed, depending on the capacity of the computer used. Users can solve this problem by reducing the representation to higher confidence type connections.

Validations page: displays a table with the pairs of interacting entities and corresponding abstract that have been already validated by the user. It permits the validation table to be saved so that validations can be loaded in other projects and shared with other users.

## 3. Results and Discussion

PESCADOR, distinctly from other co-occurrence-based text mining tools, allows selecting gene/protein co-occurrence pairs based on their relatedness to biological concepts and therefore, brings together under a common perspective protein interactions that have not been studied under the same research focus. This property can be graphically observed on the behavior of edges displayed on the global network at the PESCADOR web site. In the following paragraphs we exemplify this with two case studies.

### 3.1. Case study #1: role of protein aggregation and processing in neural disease

We analyzed a thematic selection of literature consisting of 49 abstracts related to Alzheimer's and Parkinson's diseases in the context of protein processing and aggregation surrounding the protein-protein interactions of two human proteins associated to Alzheimer's and Parkinson's disease: the amyloid beta precursor protein (Abeta, encoded by the APP gene) [26] and alpha-synuclein (encoded by the SNCA gene) [27], respectively. Alzheimer's and Parkinson's diseases share some phenotypical and clinical characteristics; formation of plaques of protein aggregates in the brain of patients is one of those common features. The question we wanted to address with this analysis is whether the Abeta and alpha-synuclein proteins are interconnected through common genes, proteins and processes relevant in the context of protein processing and aggregation. As we will discuss later, such a query cannot be easily handled with current tools for extraction of biomolecular interactions from the literature. This analysis is accessible, among other illustrative cases, from the current PESCADOR home page.

After abstract tagging, a total of 107 gene/protein terms were identified in the selected 49 abstracts. This is the most computationally demanding step typically requiring one second per abstract. However, tagged abstracts are stored making re-analysis almost instantaneous.

A total of 532 biointeraction sentences were identified, 63 of them of type-1, with 48 and 11 for the amyloid beta precursor protein (gene: APP) and for alpha-synuclein (gene: SNCA), respectively, and one more between two other proteins. When these terms and interactions are displayed graphically two large hubs appear centered on the two proteins focus of our study with one connection between them (Figure 1A). It must be noted that the structure of this network has no biological relevance but just reflects that the query focused on the two proteins at the center of each hub.

By adding a selection of concepts we can relate parts of the interaction network to molecular processes and disease names. In particular, we can observe that the terms "cleavage" and (logically) "Alzheimer" appear to be related to the APP hub, whereas "aggregation" is attached to both the APP and the SNCA hubs (Figure 1B). Examination of the type 1 interactions related to aggregation points out that Abeta (gene: APP) promotes alpha-synuclein (gene: SNCA) aggregation [28].

To search for other indicators of this possible association between Abeta and alpha-synuclein, we examined the type-2 interactions. One extra connection appears that connects Abeta to alpha-synuclein. The evidence behind this connection is linked from the Term list and corresponds to the sentence "Deposits of AMYLOID proteins, including Abeta and alpha-synuclein coexist in the brains of patients with dementia with Lewy bodies; however, it is not known how either of them interacts with tau to provoke neurofibrillary tangle formation across the tauopathies" [29] (Figure 1C). Both protein names appear in the same sentence and a biointeraction term is recognized ("interacts") but it is not between them as it refers to another protein. However, the sentence does relate the two proteins as forming part of protein deposits in a neurodegenerative disease.

In this example, PESCADOR offers an overview of terms associated to the PPI network and of their relation to disease; a user can visualize the proteins associated to those terms and eventually revisit the bibliography from which the connections were derived. Biological concept usage on literature mining could also be explored to filter large networks and display nodes connected only to desired concepts, a feature that can be used at the "Concept Report" page of PESCADOR.

### 3.2. Case study #2: literature-supported enrichment of a KEGG pathway

The annotation of pathways requires manual selection and examination of literature and extraction of relevant interactions between genes and proteins. If the pathway is large this can be time consuming, especially if active research in the topic requires constant updates. PESCADOR is especially indicated for such a task. Here we illustrate how PESCADOR can be used to expand a pre-existing already large (40 annotated genes) KEGG pathway: *Homo sapiens *pathway "Colorectal Cancer" (KEGG ID: hsa05210).

First, we selected PubMed abstracts related to the pathway's topic with the web-server MedlineRanker [21], which uses a Bayesian classifier to find literature relevant to a topic of choice based on the difference in word usage in PubMed abstracts between a training dataset and the complete Medline database. We defined the training dataset by the PubMed query "colorectal AND (cancer OR tumor OR carcinoma)", resulting in more than 67,000 abstracts. The resulting list was ranked by MedlineRanker and the top 500 PMIDs were used as input query list on PESCADOR.

We set the gene dictionary to *Homo sapiens*, and, finally, added the terms "CARCINOMA", "COLORECTAL CANCER", "HNPCC" (hereditary nonpolyposis colorectal cancer) and "TUMOUR" as biological concepts in the search. This analysis is accessible, among other illustrative cases, from the current PESCADOR home page.

The resulting network of interactions indicates the prominent focus of research on the role of beta-catenin 1 (encoded by gene CTNNB1) and their interacting partners in colon cancer. Beta-catenin 1 is part of the adherens junction protein complex, which regulates cell growth and adhesion in epithelium and is an important component of the Wnt signaling pathway. Mutations in the CTNNB1 gene or in the genes encoding proteins that interact with its protein product can result in the pathological activation of the Wnt signaling pathway, which seems to be a cause of colorectal cancer and other cancers [30].

**Figure 2:**
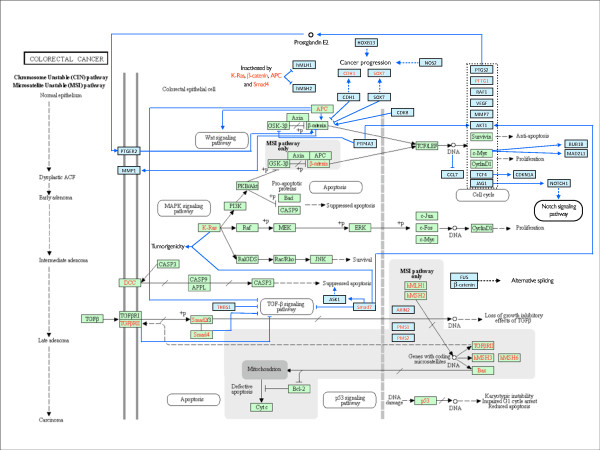
**Expanded representation of the KEGG pathway "colorectal cancer"**. The current version of the KEGG pathway "colorectal cancer" was expanded from 40 to 70 genes using PESCADOR on a selection of 500 abstracts relevant to the topic. Green boxes: genes previously annotated. Cyan boxes: genes added by our analysis. Gene names in red font: mutated in colorectal cancer.

Manual inspection of PESCADOR text-mining results allowed us to define 30 new Colorectal Cancer pathway members qualified by 55 biointeractions documented by their corresponding PMID (Additional file 1 Table S1). These new members and interactions were manually drawn to fit in the KEGG chart representing the pathway (Figure 2).

The new members added to the pathway include critical genes and their roles in colorectal cancer development have been recently established, such as the tumor suppressor E-cadherin (CDH1). CDH1 is responsible for downregulating beta-catenin and consequently diminishing cellular growth; recent studies indicate that the loss of CDH1 could therefore contribute to this pathway in human cancers [31]. Another tumor suppressor that regulates beta-catenin1 transcriptional activity is SOX7; it appears that most colorectal cancers require SOX7 inactivation in order to develop [32]. The pathways activated by mutated beta-catenin1 lead to the upregulation of several genes through the binding of TCF/LEF to specific activation sites on the DNA called TBEs. This is shown in the KEGG colorectal cancer pathway and genes such as c-Myc and Cyclin-D1 are activated in this manner. Other genes were added to the pathway as a result of this TCF dependent activation, such as MMP7, TCF4 [33], AKT1 and many others. AKT1 overexpression was recently demonstrated to be an early event in colorectal carcinogenesis and is a result of the presence of the mutated beta-catenin1 gene [34]. On the other hand, the TGFbeta pathway needs to be suppressed in order for the cancer cells to develop and not undergo apoptosis. Several proteins are directly involved in this repression and a new addition to the pathway with that particular function is SMAD7. The overexpression of SMAD7 was shown recently to block TGFbeta pathway and the function of the tumor suppressor SMAD proteins (2, 3 and 4) [35]. Blockage of the TGFbeta pathway results in cell cycle progression and growth induction. SMAD7 also interacts with AKT1 and leads to induction of ASK1, increasing cell survival and blocking apoptotic pathways, respectively. Smad7 also cooperates with activated Ras and induces tumorigenicity [35].

Thus, by using abstracts selected from PubMed through a thematic query of interest, PESCADOR provides a tool for the extraction of known regulations associated to a specific process, unlike other currently available text-mining tools.

### 3.3. Evaluation of PESCADOR on an instance level

In order to evaluate the efficiency of PESCADOR in recognizing individual PPIs we have used a PPI dataset extracted from the AIMed corpus [36]. This dataset contains 307 human PPIs manually extracted from 174 PubMed abstracts. These 174 abstracts were analysed by PESCADOR. Then, the PPIs from AIMed and PESCADOR (types 1, 2 and 3) were pooled and the resulting set was manually evaluated (Additional file 2 Table S2).

The number of AIMed interactions used for the comparison was reduced from 307 to 222, to exclude those that PESCADOR is not expected to detect by definition: self-interactions (homo-dimers) and interactions where one of the partners is defined by a symbol that does not correspond to a protein or a gene (for example, complexes, mutants or protein fragments).

Surprisingly, we could manually confirm the correctness of only 201 of the 222 instances of the AIMed dataset upon reading the corresponding abstracts. Indeed, PESCADOR identified 24 true PPIs that were not considered in the AIMed data. These were pooled with the 201 true positives from AIMed and the recall and precision of AIMed and PESCADOR were then computed with respect to this pooled set of 225 true positive PPIs (Table 1). The results indicate that type 1 interactions from PESCADOR offer high precision (80%) for a low recall (25%), whereas less restrictive interaction types permit better recall for lower precision.

**Table 1:** Recall and precision of AIMed and PESCADOR.

	Recall	Precision
AIMed	201/225 (89%)	201/222 (91%)
PESCADOR (type 1)	56/225 (25%)	56/70 (80%)
PESCADOR (type 2)	76/225 (34%)	76/109 (70%)
PESCADOR (type 3)	90/225 (40%)	90/136 (66%)

The relatively low figures of recall were mostly due to problems in the entity recognition machinery of PESCADOR. For example, many gene and protein names in the abstracts were absent from the dictionary used by PESCADOR making their detection by PESCADOR impossible. The fact that the AIMed dataset regards abstracts from the years 1998-1999 made this an important factor; since then gene names are better normalized. In addition, the engine used by PESCADOR to recognize bioentity names, NLProt, would sometimes fail to identify correct names. Finally, exclusion of sentences with four or more names was a further reason for missing a PPI. Considering only sentences favorable to name detection by PESCADOR at these three levels increased recall up to 95% (Table 2), suggesting that enhancing name detection is a possibility to improve the efficiency of PESCADOR.

**Table 2:** Influence of entity name recognition on the recall of PESCADOR

	All sentences	Both names in dictionary	NLProt detected both names	Not complex
Type 1	56/225 (25%)	56/169 (33%)	56/137 (41%)	56/95 (59%)
Type 2	76/225 (34%)	76/169 (45%)	76/137 (55%)	76/95 (80%)
Type 3	90/225 (40%)	90/169 (53%)	90/137 (66%)	90/95 (95%)

## 4. Conclusions

PESCADOR is available at http://cbdm.mdc-berlin.de/tools/pescador/. The system is platform independent and can be accessed from every common web-browser running the Java Runtime Environment (JRE) plug-in. PESCADOR was developed with an emphasis in the graphical representation of biointeractions extracted from the literature and in their association to user-defined concepts.

Three commonly used platforms that, like PESCADOR, perform text mining analysis based on co-occurrence of protein terms and provide a graphical view of such relations are STRING [15], iHOP [14] and AliBaba [16]. However, we must note that comparing these systems with PESCADOR is relatively difficult considering that they have been designed with very different goals. Most importantly, these platforms differ in the types of inputs and outputs, provide different querying mechanisms, and have different information extraction mechanisms. These and other important differences are summarized in Table 3. Shortly, iHOP navigates the co-occurrence network among terms in the literature but it does not display it. Both STRING and iHOP use text mining of the whole PubMed database. PESCADOR instead uses pre-selected abstracts to infer networks; each edge of the network (an interaction between a pair of entities identified in an abstract) can be manually validated. AliBaba has an advanced graphical interface and, like PESCADOR, uses PubMed abstracts as input, can extract biointeractions with confidence degrees, and allows editing the graph of co-occurrences. However, according to the help information of the current version (1.5 (2009-12-18)) it is running on a PubMed version from 2006, which might limit its usefulness as a discovery tool. In summary, these tools allow analyses that have certain similarities but, among them, PESCADOR is unique in its focus on revealing the processes related to the interaction network.

**Table 3:** Feature comparison among PESCADOR, iHOP, STRING and AliBaba.

Feature	PESCADOR	iHOP	STRING	AliBaba
Input	PubMed (user selection or query)	Protein/Gene ID	Protein/Gene ID	PubMed (query)

Biological concepts	Customized	Fixed	Fixed	Fixed

Literature corpus	Selected PubMed	Whole PubMed	Whole PubMed	Selected PubMed

Co-occurrence context	Sentence/whole abstract	Sentence	Whole abstract	Sentence

Target protein display filters	Biological concept; Biointeraction term; Co-occurring pair; Co-occurrence structure.	Co-occurring pair.	Co-occurring pair; Confidence; Degree.	Co-occurring pair; Confidence; Degree.

Manual validation of co-occurring pairs	Yes	Yes	No	No

Co-occurrence validation sharing among users	Yes	No	No	No

Network building	Automatically on-the-fly	Manually stepwise	Automatically on-the-fly	Automatically on-the-fly

In the near future we plan to implement the text-mining methods used by PESCADOR server as a set of Web Services, permitting the integration of our pipeline on other pipelines aiming at literature analysis. Another future goal is to permit the simultaneous use of dictionaries of gene/protein names from multiple organisms; by doing that, we expect to filter from the literature important co-occurrences of gene/proteins from interacting organisms under a determined concept, such as host-pathogen molecular interactions under the course of a determined infection/disease.

Finally, the benchmark of PESCADOR on the AIMed dataset suggested that a large number of missed PPIs are due to the failure to recognize entity names (Table 2), which is dependent on LAITOR, the text-mining engine of PESCADOR. To approach this problem, we intend to take advantage of recent developments in the field of information retrieval to improve LAITOR. We could try BANNER [37] for the recognition of genes, MetaMap for concepts [38], or kernel methods for relation extraction [39,40]. Any improvements in LAITOR should result in an ensuing improvement of PESCADOR.

## 5. Competing interests

The authors declare that they have no competing interests.

## 6. Availability and requirements

Project name: PESCADOR

Project home page: http://cbdm.mdc-berlin.de/tools/pescador/

Operating system: Platform independent

Programming language: PHP

Other requirements: none

License: none

Any restrictions to use by non-academics: none

## 7. Authors' contributions

ABS developed PESCADOR. ABS and MAA designed the functionality of PESCADOR. JFF, ERD, FS and MO tested PESCADOR and prepared examples of its application. ABS and MAA wrote the manuscript. All authors read and approved the final manuscript.

## Supplementary Material

Additional file 1 **Table S1: Pairs of genes/proteins (Term 1, Term 2), biointeractions, and the literature (PMID) used as evidence to add 30 new members to the KEGG pathway: Homo sapiens pathway "Colorectal Cancer" (KEGG ID: hsa05210)**. Click here for file

Additional file 2 **Table S2: Detailed benchmark of PESCADOR on an instance level using the AIMed dataset**. The data is arranged in rows for each PPI and by PMID. Columns are **Term1 from AIMed **and **Term2 from AIMed **for AIMed PPIs, **why filtered out from AIMed**, for AIMed PPIs that would not be included in the analysis (e.g. because they were duplicated), **Term 1 from PESCADOR **and **Term 2 from PESCADOR **for PESCADOR terms for PPIs, **found in AIMed **for whether the PPI was detected by AIMed, **PESCADOR Type 1, Type 2**, and **Type 3 **for whether the PPI was detected by PESCADOR in one of these levels (only strictest one is indicated), **found by manual curation**, indicating if the PPI is real according to our evaluation, **evidence sentence **indicates the sentence where the PPI could be found, **note **indicates extra explanations of our analysis, and **cause of PESCADOR failure **gives one of three reasons of why a true PPI detected by AIMed was not found by PESCADOR: NLProt fail, name not in dictionary, or sentence too complex. Click here for file
