# Supplementary material for: PESCADOR, a web-based tool to assist text-mining of biointeractions extracted from PubMed queries
Source: BMC Bioinformatics. 2011 Nov 9;12:435. doi: 10.1186/1471-2105-12-435 (PMC3228910; doi:10.1186/1471-2105-12-435)
Supplement: Additional file 1 — Table S1: Pairs of genes/proteins (Term 1, Term 2), biointeractions, and the literature (PMID) used as evidence to add 30 new members to the KEGG pathway: Homo sapiens pathway "Colorectal Cancer" (KEGG ID: hsa05210). [file 1471-2105-12-435-S1.PDF]

Term 1	Biointeraction	Term 2	PID
APC mutated	perturbing the function	hMLH1	12142355
APC mutated	perturbing the function	hMSH2	12142355
APC mutated	perturbing the function	TGF-B signaling pathway	12142355
APC mutated	activates	n.a.	10772417
B-Catenin (CTNNB1)	activates	TCF	15888491
B-Catenin (CTNNB1)	increases transcription	c-Myc (MYC)	16998321
B-Catenin (CTNNB1)	upregulates	PTGS2	12566320
B-Catenin (CTNNB1)	induces overexpression	PTTG1	16705313
B-Catenin (CTNNB1)	increases transcription	VEGF	16998321
B-Catenin (CTNNB1)	induces	JAG1	19325125
B-Catenin (CTNNB1)	induces	RAF1	16478791
B-Catenin (CTNNB1) / FUS	induces	alternative splicing	16230076
B-Catenin mutated (CTNNB1 mutated)	enhances activation	AKT1	15888491
B-Catenin mutated (CTNNB1 mutated)	perturbing the function	hMLH1	12142355
B-Catenin mutated (CTNNB1 mutated)	perturbing the function	hMSH2	12142355
B-Catenin mutated (CTNNB1 mutated)	increases expression	MMP7	12481159
B-Catenin mutated (CTNNB1 mutated)	epigenetic silencing	SOX7	18819930
B-Catenin mutated (CTNNB1 mutated)	increases expression	TCF4	12481159
B-Catenin mutated (CTNNB1 mutated)	downregulates	CCL7	11118053
c-Myc (MYC)	activates	BUB1B	17297307
c-Myc (MYC)	activates	MAD2L1	17297307
CDH1	inhibts	B-Catenin (CTNNB1)	11381089
CDK8	activates	B-Catenin (CTNNB1)	19790197
HOXB13	loss induces	cancer progession	15928669
JAG1	induces	Notch1	19325125
KRAS mutated	perturbing the function	TGF-B signaling pathway	12142355
KRAS mutated	perturbing the function	hMLH1	12142355
KRAS mutated	perturbing the function	hMSH2	12142355
MMP1	transactivates	B-Catenin (CTNNB1)	12185585
NOS2	induces	cancer progression	11935128
PMS1	is mutated	n.a.	9438104
PMS2	is mutated	n.a.	9438104
PTGER2	activates	B-Catenin	16998321
PTP4A3	activates	AKT1	17409395
PTP4A3	inactivates	GSK3B	17409395
PTP4A3	activates	AKT1	17409395
PTP4A3	downregulates	GSK3B	17409395
Smad4 mutated	perturbing the function	hMLH1	12142355
Smad4 mutated	perturbing the function	hMSH2	12142355
Smad4 mutated	perturbing the function	TGF-B signaling pathway	12142355
Smad4 mutated	inactivates	TGF-B signaling pathway	12618756
Smad7 mutated	overexpression inhibits	TGF-B signaling pathway	18781153
Smad7 mutated	activates	akt1	18781153
Smad7 mutated	activates	ASK1	18781153
TCF	activates	AKT1	15888491
TCF	activates	PTTG1	19394332
TCF	activates	TCF4	19394332
TCF	activates	MMP7	19394332
TCF4	regulates	p21 (CDKN1A)	19394332
TCF4	activates	CDKN1A	19394332
THBS1 mutated	inactivates	TGF-B signaling pathway	18425817
PTGS2	generates	Prostglandin E2	16998321
Prostglandin E2	induces	PTGER2	16998321
Notch1	activates	Notch signaling pathway	19325125
Smad 7 / K-ras	induce	tumorigenicity	18781153
